# Flow-Field Inference for Turbulent Exhale Flow Measurement

**DOI:** 10.3390/diagnostics14151596

**Published:** 2024-07-24

**Authors:** Shane Transue, Do-kyeong Lee, Jae-Sung Choi, Seongjun Choi, Min Hong, Min-Hyung Choi

**Affiliations:** 1 Department of Computer Science and Engineering, University of Colorado Denver, Denver, CO 80204, USA; shane.transue@ucdenver.edu (S.T.); min.choi@ucdenver.edu (M.-H.C.); 2 Department of Software Convergence, Soonchunhyang University, Asan 31538, Republic of Korea; 3 Department of Internal Medicine, Cheonan Hospital, College of Medicine, Soonchunhyang University, Cheonan 31151, Republic of Korea; 4 Department of Otolaryngology-Head and Neck Surgery, Cheonan Hospital, College of Medicine, Soonchunhyang University, Cheonan 31151, Republic of Korea; 5 Department of Computer Software Engineering, Soonchunhyang University, Asan 31538, Republic of Korea

**Keywords:** CO_2_ pulmonary evaluation, non-contact pulmonary measurement, flow field interpolation, UNET flow prediction, pulmonary diagnostics

## Abstract

Background: Vision-based pulmonary diagnostics present a unique approach for tracking and measuring natural breathing behaviors through remote imaging. While many existing methods correlate chest and diaphragm movements to respiratory behavior, we look at how the direct visualization of thermal CO_2_ exhale flow patterns can be tracked to directly measure expiratory flow. Methods: In this work, we present a novel method for isolating and extracting turbulent exhale flow signals from thermal image sequences through flow-field prediction and optical flow measurement. The objective of this work is to introduce a respiratory diagnostic tool that can be used to capture and quantify natural breathing, to identify and measure respiratory metrics such as breathing rate, flow, and volume. One of the primary contributions of this work is a method for capturing and measuring natural exhale behaviors that describe individualized pulmonary traits. By monitoring subtle individualized respiratory traits, we can perform secondary analysis to identify unique personalized signatures and abnormalities to gain insight into pulmonary function. In our study, we perform data acquisition within a clinical setting to train an inference model (FieldNet) that predicts flow-fields to quantify observed exhale behaviors over time. Results: Expiratory flow measurements capturing individualized flow signatures from our initial cohort demonstrate how the proposed flow field model can be used to isolate and analyze turbulent exhale behaviors and measure anomalous behavior. Conclusions: Our results illustrate that detailed spatial flow analysis can contribute to unique signatures for identifying patient specific natural breathing behaviors and abnormality detection. This provides the first-step towards a non-contact respiratory technology that directly captures effort-independent behaviors based on the direct measurement of imaged CO_2_ exhaled airflow patterns.

## 1. Introduction

Non-contact pulmonary evaluation presents a promising method for capturing natural breathing behaviors that are normally altered when using conventional diagnostic methods such as spirometry [1,2,3], plethysmography [4,5], and capnography (end-tidal CO_2_) [6,7,8]. While many of these pulmonary diagnostics provide highly accurate results, they also modify the natural breathing behavior by forcing unnatural posture, breathing through tubes that restrict flow, alter posture, and increase physiological effort during monitoring. This leads to effort-dependent evaluation of pulmonary behavior, which can alter the interpretation of subtle condition traits that can be detected through continuous non-contact evaluation. Unlike many new approaches to pulmonary monitoring that attempt to utilize wearable devices to infer breathing behaviors, the direct analysis of exhaled airflow cannot be captured through these methods. Rather, they rely on indirect measures of related physiological signals that provide a basis for evaluating pulmonary function. Additionally, advanced forms of respiratory diagnostics include X-ray Computed Tomography (CT) [9,10], Magnetic Resonance Imaging (MRI) [11], and Positron emission tomography (PET) scans [12]; however, these advanced scans have limited availability and incur procedures that are warranted once chronic conditions become serious enough to require detailed evaluation. As an alternative to these existing methods, we present a non-contact approach to pulmonary behavioral evaluation aimed at identifying unique characteristics directly observed expiratory behaviors through thermal CO_2_ imaging [13,14]. The proposed method sits in between existing wearable, tube-based, and advanced scan solutions, presenting an approach that can provide a basis for pre-screening and anomalous behavior detection.

**Figure 1:**
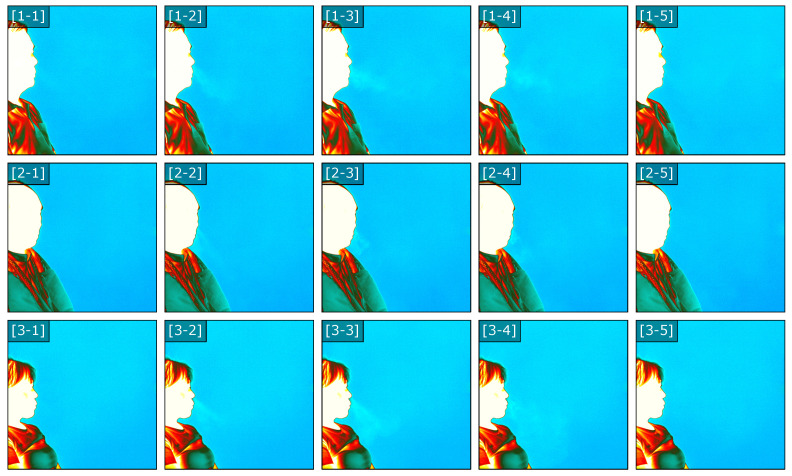
Flow sequences from three selected subjects illustrating unique exhale patterns. Each sequence presents static states of the captured infrared thermal CO_2_ flow for a single exhale episode for each of the selected subjects. Each sequence presents five (h=512, w=640) frames for each subject that exemplify the flow of each patient’s exhale pattern.

In this work, we present a method for utilizing imaged exhale behaviors captured through thermal CO_2_ flow fields to directly measure exhale flow and form unique characteristic waveforms that uniquely describe individualized pulmonary signatures (Figure 1). By capturing the unique behaviors from 20 different subjects, we identify how a flow inference model can be utilized to extract, filter, and interpolate exhale flow characteristics. This enables us to define and characterize how individual posture and effort contribute to the exhale behaviors that give insight into natural breathing. Our goal is to provide a basis for identifying how these unique traits can be used to create insight into how natural breathing reflects conditions including Chronic Obstructed Pulmonary Disease (COPD) [15,16], asthma, Acute Respiratory Distress Syndrome (ARDS) [17,18,19], Obstructive Sleep Apnea [20,21], and Respiratory Syncytial Virus (RSV) [22]. In our approach, we provide a pipeline that translates recorded or real-time sequences of thermal images into flow-field estimates that can be used to measure and uniquely identify individualistic traits of expiratory behavior. This includes: (1) using facial tracking to (2) performing dense, detailed flow analysis, enabling us to (3) quantify and model flow behaviors within an open clinical environment. We introduce a new Convolutional Neural Network architecture: *FieldNet*, based on architectures similar to U-Net [23] and FlowNet [24] to predict exhale flow fields. The proposed model operates on vector fields to perform flow-field inference to isolate and extract exhale flow behaviors that can define individualized pulmonary traits.

While capturing detailed exhale behaviors through a non-contact method presents unique advantages, there are several technical challenges that make this approach competitive within the existing field of pulmonary diagnostic technologies. The challenges of using a vision-based approach to measuring exhale behaviors are greatly influenced by three primary factors: (1) subject movement must be accounted for to provide continuous and reliable monitoring, (2) capturing flow behaviors of the thermal signature requires sensitive imaging hardware and detailed flow tracking, and (3) airflow behaviors are mixed with general air movements as well as backgrounds that interfere with flow isolation. To address the first requirement, we adopt facial tracking and minimize the impact on computed flow fields. Second, we leverage Mid Wavelength Infrared Camera (MWIR, 640×512@25 Hz) thermography paired with a spectral band-pass filter (3–5 μm) and parallel optical flow tracking [25] for generating dense 2D flow-fields. Finally, we leverage the ability of a flow-field inference model to extract and isolate flow behaviors to create exhale measurements. The proposed flow model provides three primary functions: (1) the ability to segment and separate airflow behaviors for measurement, (2) the ability to predict interpolated flow-fields, and (3) isolate exhale behaviors from flow fields to improve signal extraction.

The aim of this study is to capture and quantify individualized traits of natural exhale behaviors to measure pulmonary function and isolate instances of anomalous breathing. To accomplish this, we provide three primary contributions as the objectives of the study: (1) track and capture exhale behaviors from various subjects to identify inter-subject exhale behavioral variance, (2) train a convolutional deep neural network to isolate exhale flow behaviors from open-air recordings, and (3) generate and analyze expiratory waveforms regarding the unique signatures of individualized pulmonary behaviors to identify possible anomalies within exhale episodes using a model trained on expected versus abnormal exhale patterns.

## 2. Related Work

Numerous respiratory monitoring devices and pulmonary diagnostic technologies are prominent in modern healthcare [26,27,28,29,30]. These typically fall within four primary modalities: (1) tube-based solutions that provide accurate airflow measurements, (2) advanced scanning technologies that include X-ray and CT scans [19,31], (3) wearable solutions that are worn continuously such as transducer or accelerometer belts [32,33] and health monitors [34], and (4) non-contact technologies such as imaging [35,36,37,38,39], wireless [40,41], and smartphone technologies [40]. Tube-based solutions provide the most accurate measurements that include respiratory rate (BPM), flow (L/s), volume (*L*) which are formulated into Pulmonary Function (PFT) Testing measures (VC FVC, ERV, TV, etc.) [15,42]. This segment contains many of the primary pulmonary diagnostics utilized in clinical settings including spirometry [1,2,3], plethysmography [4,5], and capnography [6,7,8]. If monitored pulmonary conditions indicate severe progression, advanced scanning technologies such as X-ray and CT scans may be used to identify interstitial lung abnormalities (ILAs), regional ventilation, perfusion, and gas exchange [43,44]. While these scans provide some of the most accurate physiological measures, they are also costly and largely restricted to clinical use. Looking beyond clinical pulmonary diagnostics, wearable devices focus on the translation of chest movements or other physiological signals into pulmonary metrics. The most common forms of wearable devices that measure breathing rate and volume are transducer and accelerometer belts [32,33]. These devices translate chest movements and expansion as a correlated function that describes respiratory behaviors. Improvements in these devices have lead to accurate measurements of behaviors and are commonly used in exercise and sleep studies [20,21]. The benefit of wearable devices is that they provide continuous signals, but accuracy is generally reduced by sensor placement and movement. Smart watches and fitness trackers also include biometric readings related to respiration; however, while these devices are accurate for heart rate, they still have to infer respiratory characteristics through other physiological signals. Recent methods have utilized machine learning to infer these measures from reliable sources such as heart rate [45]. The majority of vision-based methods for respiratory analysis have leveraged the relationship between observable chest movements with the cyclical breathing patterns [46]. Many of these approaches utilize depth imaging or color images to identify shifts in movement that correspond to the observable respiratory movements [47,48]. The weakness of these approaches is in the correlation between the observed movement and how the measurement is translated into a quantitative signal representing the inspiratory and expiratory behaviors. While this correlation is closely related to the premise used for transducer and accelerometer respiratory belts, vision-based methods are typically less accurate due to the unbound relationship between camera and subject [49,50]. The second common form of vision-based approaches to respiratory monitoring utilize infrared imaging; however, these approaches focus on the detection of temperature differentials on the face (nostrils) to identify breathing behaviors [49,51,52,53]. These approaches operate in a similar fashion to thermistors that are used in sleep studies, but are largely limited to measuring respiratory rate. Vision-based models have been proposed for identifying, segmenting, and extracting gaseous flow behaviors from images and image sequences for other applications. The detection of plume behaviors for automated smoke segmentation [54] and vapour formations have been approached for a wide variety of applications including forest fire tracking [55], automated fire detection [56,57], and cloud movement tracking [58,59].

## 3. Materials and Methods

**Figure 2:**
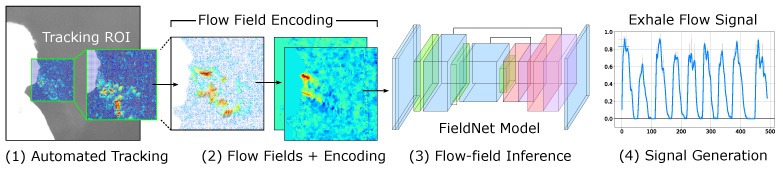
Approach overview. Facial tracking within thermal sequences capturing exhale behaviors is used to localize the ROI for which 2D flow fields are generated. These fields are then encoded and used to train the proposed *FieldNet* architecture for predicting flow fields. These fields and then used to generate signals representing captured exhale behaviors.

The objective of vision-based thermal flow expiratory modeling is to identify, track, and extract meaningful respiratory traits that uniquely describe pulmonary function. The unique contribution to this approach is that imaging expiratory behaviors can provide a detailed visual of natural breathing which, characteristic traits that model individualized traits. By directly capturing exhaled airflow, we can identify characteristic traits of an individual’s subconscious breathing pattern that is not directly altered by the monitoring method. To achieve this, we present an automated pipeline that allows the monitored subject to relax and breathe naturally for a short period of time while capturing their unique breathing signature. During this monitoring phase, we record the stream of thermal images that capture exhale behavior. These image sequences are then provided to a processing pipeline that: (1) tracks the subjects movement and localizes the Region-of-Interest (ROI) to the nose-mouth sub-image, (2) computes a fine-grained dense optical flow to generate flow-fields that capture expiratory behavior, (3) encodes this information to train a generative flow-field model to predict flow behaviors, that can then be used to (4) generate waveform representations of the observed exhale behaviors. An overview of the implemented data processing pipeline is shown in Figure 2.

The foundation of the approach is based on the creation of image datasets that contain exhale behaviors from various subjects that are used to train and evaluate the proposed FieldNet model and generate individualized respiratory measurements. This process is divided into four primary steps that include: data acquisition, ROI selection and tracking, flow field generation, and training data collation. The resulting trained model is then used to predict flow fields with isolated exhale vectors that can be used as a measurement to evaluate airflow. This provides the ability to use the model on each of the 20 subjects included in the initial cohort to generate non-contact respiratory signals that exhibit physiological differences in the highly individualistic measurement waveforms. This provides a basis for observing natural breathing behaviors as a pulmonary diagnostic that can be used to identify unique characteristics of respiratory traits.

### 3.1. Data Acquisition

The data collection procedure was performed within an outpatient clinical setting where each subject was recorded for approximately five minutes. Data acquisition was performed at Soonchunghyang University Cheonan Hospital, IRB (no. 2023-10-012). The basic arrangement provided: (1) tripod mounted camera approximately 1.5 [m] from the subject, (2) seating position perpendicular to the camera, parallel to the imaging plane, and (3) the clinical tech and recording software running on a locally connected computer. Given this setup, turbulent exhale flows are captured and projected from the 3D space between the camera and background and projected to a 2D flow representation that is imaged by the thermal camera. Thermal images are captured and stored as raw 16-bit (0-$2^{16}$-1) *count* (sensor activations to pixel intensity) image sequences stored in contiguous binary field sequence files. These pixel values typically range between 5000–9000 counts an interior room at 22 °C. The content of the image can be heavily modified by the selected range of raw pixel values that are normalized (p∈[0,1]); therefore, we form two algorithms for: (1) a normalization that prioritizes tracking and (2) a normalization range that emphasizes observable exhale flow. For tracking, the normalization is set to constant at the high and low values of the image to provide clear feature-points that can be tracked to enable the automated facial tracking: $\left[F_{c},I_{max}\right]\to \left[0,1\right]$, where $F_{c}$ is the count corresponding to the face temperature. For the exhale normalization we sample the background values and identify this as the general ambient count ($A_{c}$) within an ROI of the image and identify high bound as the face temperature $F_{c}$. Since the exhale intensities will fall between these values, this normalization is defined as: $\left[A_{c},F_{c}\right]\to \left[0,1\right]$. This creates two parallel uses of each image contained within the recording allowing for subject movements to be tracked to dynamically adjust the ROI used to capture exhale flow behaviors.

### 3.2. Automated Tracking

The imaging process captures the subject as well as the surrounding area, with a portion of the image reserved to capture exhale behaviors within the tracked ROI. The immediate challenge for accurately capturing a subject’s breathing behavior is closely tracking the nose-mouth region from the profile view while minimizing the impact of subject movement on the process of generating the flow field. As the ROI moves, this can introduce uniform shifts within the flow field that contribute to error within the measurement of the exhale behavior. To address this, we employ automated tracking that: (1) accurately tracks the nose-mouth region and (2) minimizes the per-frame jump required to maintain a constant tracking region that best captures the exhale behavior. Since the outcome of the tracking is an ROI shift, the offset for the computation of the flow field can be addressed through an inverse of the transformation. This is utilized to eliminate the shift of the ROI that would be exhibited within the flow field for subsequent frames. While facial tracking in infrared images has been studied [60], these methods are not directly applicable to profile tracking restricted to the nose-mouth region. Similarly, Haar cascades can be adopted to infrared imaging [61]; however, this approach is not well suited for profile facial tracking in this application due to translational jumps during tracking.

**Figure 3:**
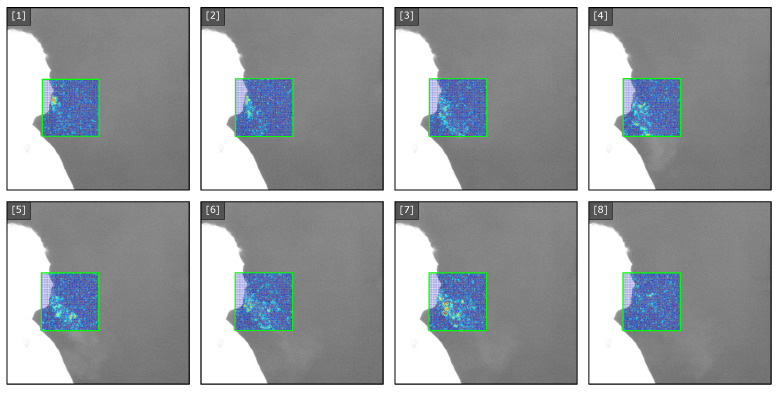
Tracking region for select frames 1–8. Optical flow is utilized to identify the correspondence and movement between subsequent frames of the tracked region. This fixed window is tracked throughout all frames of the sequence. The flow vectors are displayed using the real-time approximation (stride = 2 + line segment rendering) for images of size (h=512, w=640).

To achieve accurate tracking within the recorded sequence, we utilize a fixed sub-region of interest of fixed size that correlates to the the input dimensionality of the flow model. This sub region window will be used to capture the imaged exhale flow as the thermal distribution of the flow over time in contrast to the static background. First, we identify the nose/mouth region of the subject and then define the fixed size sub-region within the initial frame. As each frame is processed, key-points within the sub-region corresponding to features on the subject’s face are identified. Lucas-Kanade optical flow [62] is then used to estimate the translation vectors of these key-points to update or shift the sub-region during the subsequent frame. To provide real-time feedback, the proposed parallel dense optical flow is used to compute the flow vectors within the sub-region corresponding to the exhale behavior. The image sequence in Figure 3 illustrates the captured image, the sub-region, and the magnitude of the flow vectors within the region.

**Figure 4:**
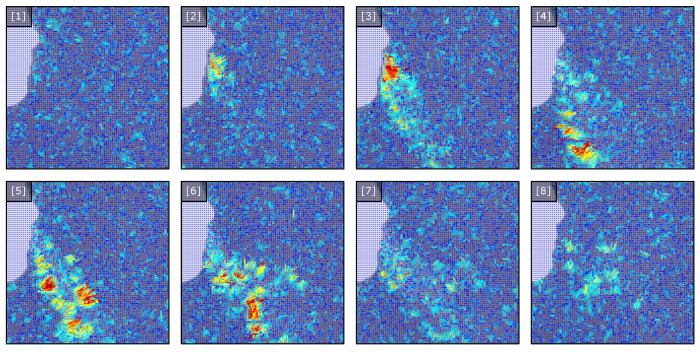
Tracked flow sequence for select frames 1–8. The tracked sub-region of the exhale is recorded for both training and inference datasets. Flow vector magnitude and direction is displayed using line segments (stride = 2) for the tracked sub-region. Color indicates flow vector magnitude.

Within the tracked sub-region, the flow is computed using a parallel implementation of a noise-sensitive dense optical flow and superimposed on the image used to provide feedback during clinical recording. The rendering is based on drawing scaled line segments representing the flow field with a stride of s=2 and color-mapped flow vector. This provides an approximation of the vector field being computed from the recorded frames. The image sequence in Figure 4 illustrates the states of the vector field representing the apparent exhale flow for eight consecutive images. Based on the progression of the observable exhale flow, the resulting flow field captures the magnitude of the localized flow as the image intensities shift between frames.

The apparent flow identified within the tracked ROI provides the basis of the measurements used to quantify and analyze exhale behavior. Exhale flow fields are computed between subsequent frames within the collected sequences and are then used to train the FieldFlow model. The process of generating the fields is based on performing dense optical flow [25] that creates a 2D vector field of the contribution of the gradient shift in image intensity represented by vector direction and magnitude. The iterative scheme of the optical flow algorithm is then used to detect minute directional flow changes observed within the imaged exhale pattern to both separate exhale flow from background air movements and provide the basis for quantifying the changes in observable flow patterns between subsequent frames.

### 3.3. Flow Field Generation

Observable flow contained within the recorded infrared image sequences is computed as the optical flow of the shifts of intensity obtained from image pairs [25,63]. To generate flow sequences from each recording, we define the flow as the *apparent* shifts in intensity obtained from iteratively solving for the brightness and smoothness constraints of the optical flow formulation [25]. This computes the flow field ${\vec{F}}_{t}\left(i,j\right)$ between consecutive normalized image frames $I_{t}$ and $I_{t+1}$ based on the minimization form of the brightness and smoothness constraints as shown in Equation (1).

(1) $$\min\;\int \int \;{\left(\nabla I\cdot v+\frac{\partial I}{\partial t}\right)}^{2}+\alpha (∥\nabla v_{x}{∥}^{2}+∥\nabla v_{y}{∥}^{2})\;dxdy$$

where $\vec{v}=\left(v_{x},v_{y}\right)$ represents the flow vector at each pixel correlated between the input images $I_{t}$ and $I_{t+1}$ and the scalar α specifies the contribution of the gradient magnitudes. To ensure that small fluctuations in flow are maintained within the generated flow field, α is reduced to small values (α=0.15). This ensures that intensity shifts close to the noise floor are counted as valid contributions to the observable exhale. The iterative implementation is based on the computation of two updated flow images $U_{i}$ and $V_{i}$ representing the *x* and *y* derivative images respectively at *i* iterations. The following iterative scheme presented in Equations (2) and (3) are used to update the derivative images for *n*-iterations.

(2) $$v_{x}^{i+1}={\bar{v}}_{x}^{i}-\left[\frac{\frac{\partial I}{\partial x}{\bar{v}}_{x}^{i}+\frac{\partial I}{\partial y}{\bar{v}}_{y}^{i}+\frac{\partial I}{\partial t}}{\alpha +{\frac{\partial I}{\partial x}}^{2}+{\frac{\partial I}{\partial y}}^{2}}\right]\frac{\partial I}{\partial x}=U_{i}$$

(3) $$v_{y}^{i+1}={\bar{v}}_{y}^{i}-\left[\frac{\frac{\partial I}{\partial x}{\bar{v}}_{x}^{i}+\frac{\partial I}{\partial y}{\bar{v}}_{y}^{i}+\frac{\partial I}{\partial t}}{\alpha +{\frac{\partial I}{\partial x}}^{2}+{\frac{\partial I}{\partial y}}^{2}}\right]\frac{\partial I}{\partial y}=V_{i}$$

The spatial derivatives ∂I/∂x and ∂I∂y are approximated using a Sobel kernel [64]. This results in the computation of the two channel flow field with ∂I/∂x and ∂I/∂y derivatives stored as a (h,w,2) tensor that defines the flow field ${\vec{F}}_{t}\left(i,j\right)$ after *n* iterations. The resulting form of this tensor is shown in Equation (4). These flow frame instances will then be used to create the training dataset used to train the flow interference model.

(4) $${\vec{F}}_{t}\left(i,j,0\right)=v_{x}^{n}\;{\vec{F}}_{t}\left(i,j,1\right)=v_{y}^{n}$$

**Figure 5:**
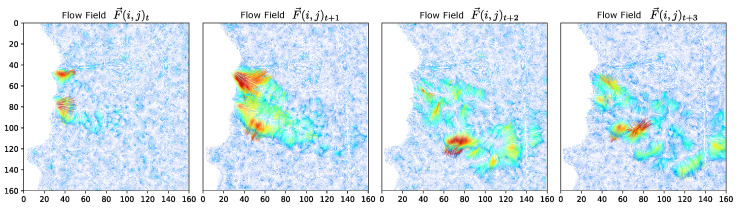
Illustration of the flow sequence vector fields for one exhale sequence. The sequence is illustrated by the flow fields for i%n frames where n=3. The flow is characterized by the initial release and dissipation of the exhale, illustrated as a function of magnitude.

While numerous advances have been proposed for the prediction of optical flow fields, the implementation is designed for real-time feedback within the clinical setting. Therefore, the our approach utilizes an optimized parallel C++/CUDA implementation compiled into a compiled Python module using pybind11 [65]. This implementation does not utilize the hierarchy from the LK method [62]. The implementation can be interchanged with numerous other modern optical flow implementations [66] as well as recent advances in adopted transformer model implementations [45]. From the implemented flow field generation, we visualize the flow sequence as shown in Figure 5.

### 3.4. Signal Generation

The objective of a respiratory diagnostic is to identify the key traits and signatures that illustrate pulmonary health and how various conditions are exhibited within recorded breathing behaviors. Typically, pulmonary behavior must be reduced to a clinically meaningful metric that adheres to existing measurements that correspond to the behavioral traits of various conditions. Since the direct presentation of 2D flow behaviors can be difficult to discern for pulmonary diagnostics, we simplify the computed flow behaviors to a one-dimensional exhale waveform that represents the overall flow over time. To do this, we assume that the gradient of flow vector contributions gradually tappers from a maximal thermal signature to zero as it dissipates. Therefore, the contributions of each flow vector within the flow field are added together to provide a one-dimensional time-series that describes the overall behavior of the captured natural breathing. To compute this for a given flow field at time *t* for the field ${\vec{F}}_{t}$, we sum the contributions from each vector within the ROI as shown in Equation (5).

(5) $$f\left(t\right)=\sum_{i=1}^{h}\sum_{j=1}^{w}\left|{\vec{F}}_{t}\left(i,j\right)\right|$$

where f(t) provides a reduction from 2D field space to a 1D time-series signal based on the sum of all contributing vector magnitudes. Each flow field provides an individual sample within this reduction, allowing us to generate a 1D time-series per sequence. While spatial information is lost, the overall characteristics of the exhale traits result in considerable differences between subjects. However, the problem with directly utilizing flow fields computed from the optical flow is that since exhaled air rapidly dissipates, vector magnitudes quickly diminish to the noise floor of the thermal cameras detection of background fluctuations. This presents a problem for obtaining clean flow behaviors that can be measured. This provides the motivation for the creation of a model that is capable of isolating the contributions of these flow vectors as they dissipate to create accurate estimates of exhale flow over time. Therefore, we present the creation of a FieldFlow model that aids in both isolation and extraction of exhale flow measurements as part of an interpolation network that enables flow-field inference to create new flow fields that: (1) eliminate background noise, (2) characterise flow behaviors, and (3) enable quantitative evaluation of respiratory behaviors.

### 3.5. Flow Field Modeling

There are numerous approaches to isolating and segmenting important information from images to obtain clinically meaningful information. U-Net architectures have been widely used in medical imaging for segmentation [23]; this architecture can also be used as a foundation for the process of extracting minute exhale behaviors that can be lost as they dissipate to background noise-levels. Additionally, FlowNet [24] based network architectures can be utilized as generative models, providing estimates of flow fields based on observed training behaviors. Based on this we present a hybrid between these network architectures that captures the intermediate behaviors of observable flow states to estimate exhale flow behaviors. To implement this, we construct a training dataset based on a subset of the recorded data formulated as contiguous flow fields that can be used to predict intermediate flow fields to: (1) isolate exhale behaviors and (2) predict filtered flow fields.

### 3.6. Flow Field Encoding

**Figure 6:**
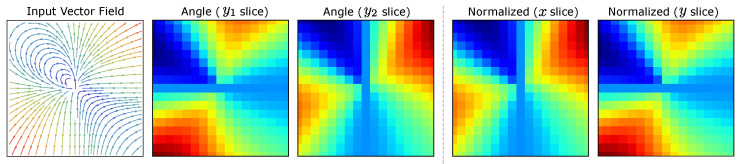
Encoding equivalency. Angular encoding (1) is equivalent to direct normalization on the bounds [0,1] when magnitude is included. Therefore the optimal encoding is the direct parallel normalization of the vector field by component. Note: Angle ($y_{1}$ slice) is equivalent to Normalized (*y* slice) and Angle ($y_{2}$ slice) is equivalent to Normalized (*x* slice).

Capturing spatially contiguous fluid flow behavioral characteristics is the primary objective of the model. This requires encoding the relative spatial distribution of angles with adjacent flow vectors to represent the change of flow over time. Projected flow fields are represented by a two-dimensional coordinate space populated by a dense set of vectors defined as $\left(x,y\right)\in R^{2}$. There are two approaches to encode this information within the input tensor of the model: (1) encode the angle-magnitude representation or (2) normalize the *x*-*y* components of the field. The angle encoding provides an intuitive approach; however, immediate problem with encoding angles directly into the spatial representation of the 2D field is that the encoding will include discontinuous angle values (0≤θ≤2π), require magnitude scaling, and still need to be normalized. A solution to this problem is to define the encoding as follows:

(6) $$\begin{array}{cc} \theta & \to \left(y_{1}=\sin\left(\theta \right),\;y_{2}=\cos\left(\theta \right)\right)\\ \left(y_{1},y_{2}\right) & \to arctan2(y_{2},\;y_{1}) \end{array}$$

where we encode the angle to a two part representation where $y_{1}=\sin\left(\theta \right)\in \left[-1,1\right]$ and $y_{2}=\cos\left(\theta \right)\in \left[-1,1\right]$. While the angle is normalized in this representation, the values still must be scaled to include the magnitude of each vector. By obtaining the maximum vector magnitude (*m*) of the field (or field sequence), a linear transformation can be used to normalize the encoding that includes both angle and norm of each vector as (θ, *n*) into the scaled pair (y1,y2)·(n/m) where the quantity (n/m)∈[0,1]. The inverse order of operations can be used to obtain the decoding of these vector where $\theta =arctan2(y_{2},\;y_{1})$ and the original $\left(x,y\right)=\left(\sin(\theta ),\;\cos(\theta )\right)\cdot \left(n\cdot m\right)$. The range of sin(θ) is based on the angle values contained within the input; therefore, to maximize the distribution of the values within the training domain, the angle-encoded values still need to be normalized. Based on the numerical domain of this encoding, we can compare this to the direct normalization of the (x,y) pair. Since each component can be scaled through a linear transformation given the maximum observed value to the range [0,1], we obtain an encoding equivalency between these representations. Encoding an asymmetrical vector field, we obtain the results shown in Figure 6 where the outcome from approach (1) becomes equivalent to (2) with the exception of the slices of the tensor being swapped.

**Figure 7:**
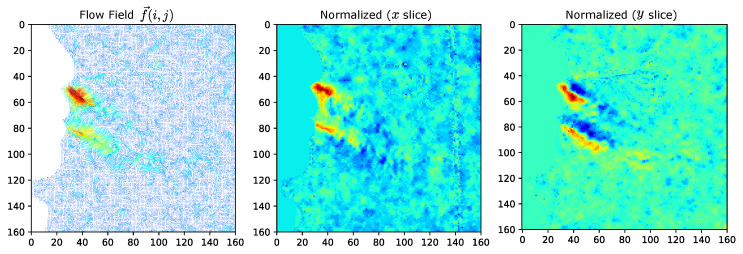
Flow field encoding visualization. The flow field (left) containing (h·w) vector pairs is encoded into the (h,w,2) tensor representing the flow between subsequent frames. The masked region of the subject contains no flow as compared to the background noise floor. The normalized slices *x* and *y* are stacked and provided as input to the FieldNet model.

This illustrates that the angular encoding is functionally interchangeable with the direct normalization with the exception of a basic flip and interchange between the two encoded channels (slices). The standard linear transformation for this normalization can be computed through an element-wise parallel process, greatly reducing the computational complexity of the encoding. This reduction is due to removing numerous trigonometric functions and required normalization if the angles within the field do not correspond to sin(θ)=1, leading to an under-utilization of the training domain range. Therefore, the encoding of the network is reduced to the parallel normalization of the field components to the interval $\left[0,n_{max}\right]\to \left[-1,1\right]$ based on the maximum vector magnitude $n_{max}$. This results in two channels of the *x* and *y* slices of the flow field as shown in Figure 7. Due to the masking introduced within tracking to remove the flow components of the face, the encoded fields will contain zero regions. While the zero regions within the encoded training dataset represents instance bias, the augmentation is used to alleviate this problem.

### 3.7. Dataset Creation

**Figure 8:**
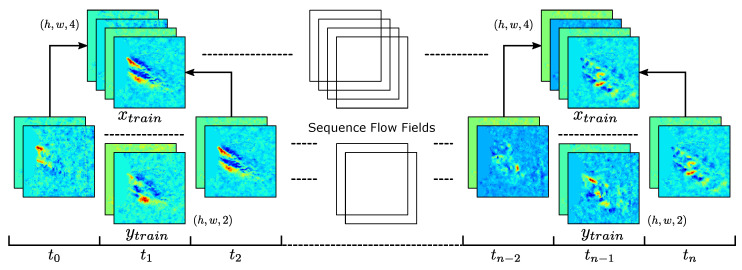
Interleaved training dataset generation. The model input $x_{train}$ is composed of two encoded flow frames from times $t_{i}$ and $t_{i+2}$, forming the input tensor of shape (h,w,4). The $y_{train}$ expected value is defined as the intermediate frame at time $t_{i+1}$ with shape (h,w,2).

Recorded sequences from 20 subjects have been used to generate flow sequences that comprise the foundation of the collected dataset that is used for both training and validation. A subset (5) of the subjects where selected to define the flow fields of the training dataset. Of each of the five training subjects, a (90/10) training/validation split was defined. The generation of a flow field sequence from a provided recording creates a collection of vector fields representing the apparent exhale flow from time $t_{0}$ to $t_{n-1}$. To provide an expected value for the intermediate flow that will be predicted by the model, there are two possible solutions: (1) the dataset can be structured to utilize intermediate frames as the expected value or (2) generate frames from a 2D fluid simulation to provide flow characteristics. Both approaches have several benefits and challenges. For one, utilizing existing data provides training data that closely matches the behavioral characteristics of expected behavior and is easily utilized. Whereas, utilizing 2D fluid flow dynamics provides correct physical behavior, but can present challenges in determining the initial conditions and progression of the simulation that closely match recorded data. In our approach, we utilize solution (1) where we select the intermediate frame $t_{it+1}$ between frames $t_{i}$ and $t_{i+2}$ as the expected ground truth that represents the intermediate flow frame. This formulates an input $x_{train}$ containing two flow frames (${\vec{F}}_{t},{\vec{F}}_{f+2}$) with a shape of (h,w,4). Based on this scheme, we define the $x_{train}$ set as every flow field at $t_{i}$ and $t_{i+2}$ and the $y_{train}$ set as the field at $t_{i+1}$. This creates an interleaved structure that defines the input and expected values of the input for training the model as shown in Figure 8.

The input of subsequent flow fields and output of the intermediate flow field creates an interpolation network; however, since the model also extracts the primary behavioral characteristics of the observed flow behavior, we utilize the model to segment flow behaviors from the surrounding environment and airflow. The dependence on subsequent frames is not a limitation; for continuous recordings of length *n*, the model can be used to predict the filtered flow fields for n−1 frames, providing a method for isolating and measuring flow patterns of the training dataset. This can be utilized to greatly reduce noise in the resulting waveforms generated for each subject.

### 3.8. Augmentation

Encoded flow fields can be directly augmented using standard image-based augmentation methods. Based on primitive transformations, these include rotation, translative shifting, and flipping. Due to the 2-channel representation of the encoded flow fields, standard library augmentation functions for images can be applied to expand the training dataset. The experimental setup within the clinical setting remained constant throughout the data collection process. Therefore, each recorded sequence places the subject to left of the each image, breathing to the right. This creates an inherent bias within the training dataset that has to be corrected. Augmentation of the training dataset includes both rotation and mirrored instances of the flow fields to account for this bias. The resulting augmentation extends the training dataset significantly; therefore, we limit the inclusion of subject training data to sub-sections of the recorded data for the subject data that is included within the training dataset. This subset of data will then be augmented prior to forming the training ($x_{train}$, $y_{train}$) and validation ($x_{test}$, $y_{test}$) datasets where the *x* set is composed of paired input fields $x_{i}=\left({\vec{F}}_{t},{\vec{F}}_{t+2}\right)$ and the expected value composing the *y* set represents the intermediary flow field $y_{i}={\vec{F}}_{t+1}$.

### 3.9. Model Architecture

**Figure 9:**
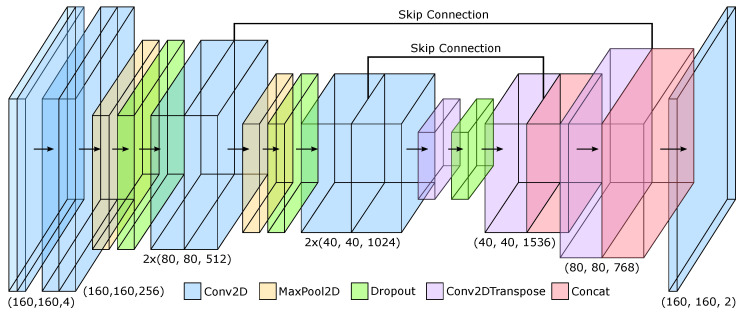
Model Architecture. The model is responsible for predicting the intermediate flow state of two flow fields stacked as an input tensor of shape (h,w,4). This is achieved by defining a UNET [23] inspired architecture that takes two encoded flow fields and predicts the encoded output flow field. Skip connections are utilized to preserve the spatial distribution of the flow throughout the network. The output defines a single intermediate flow field of shape (h,w,2).

The objective of the FieldNet model is to isolate and extract flow behaviors and minimize the contributions from background airflow and artifacts that may be introduced from background irregularities that contribute to noise. This is achieved by utilizing a convolutional network that operates on the flow fields that are generated from the parallel optical flow obtained from sequential frames. For the architecture, we adopt a U-Net convolutional model [23] that takes as input two encoded flow-fields, each of shape (h,w,2), resulting in an input tensor shape (h,w,4). The predicted output represents an encoded flow field of shape (h,w,2) which is decoded to represent the Cartesian vector field of the predicted intermediate flow state between the input fields. The implemented model architecture is shown in Figure 9 with simplified layer dimension labels. The architecture has been implemented in Keras [67] using the TensorFlow [68] back-end and trained on Google Colab Tesla T4 GPU. The model is composed of the input + 18 total layers and contains a total of 18.7 million trainable params.

Since the objective is to capture the spatial relationship between the input flow field states and the resulting prediction, *skip connections* are introduced at two levels within the architecture. This aids the model in preserving the spatial relationship between flow vectors which is critical for representing both the consistency in vector direction, but also allows the model to capture flow phenomena including vortex behaviors. Due to the range of the encoded fields [−1,1], the activation function used for all layers is the hyperbolic tangent function $tanh\left(x\right)=e^{x}-e^{-x}/e^{x}+e^{-x}$. This correlates well with the representation of the directional components of the predicted flow vectors.

### 3.10. Training

The training dataset is composed of the sequence of flow fields that are computed from the recorded thermal images from 20 subjects. To create the dataset used to train the model, we selected five of the subjects randomly and pulled sub-sequences of 500 flow frames from each of the selected subjects. This creates an initial set of 2500 flow images that are used as the training dataset that is was then interleaved following the process defined in Figure 8. This creates the input $x_{train}$ and $y_{train}$ expected values for model fit. For model loss we use standard Mean Squared Error (MSE). The selected model optimizer is Adam [69] with a learning rate of $1.0\times 10^{-3}$. For training, we use a batch size of 64 for 16 epochs. The dropout rate is 0.025 = 2.5% for all dropout layers.

### 3.11. Flow-Field Interpolation

The trained model will be used to segment the observable flow movements and generate the resulting exhale flow signal. This is based on running the model on each subject’s recorded sequence. The model will provide the segmented flow fields as a function of the current and next flow fields computed using optical flow. To obtain the interpolation prediction provided by the trained model, we provide the model subsequent flow frames as the current field ${\vec{F}}_{t}$ and the next field ${\vec{F}}_{t+2}$ to estimate the flow between these two captured states as the predicted flow field ${\vec{F}}_{t+1}$.

**Figure 10:**
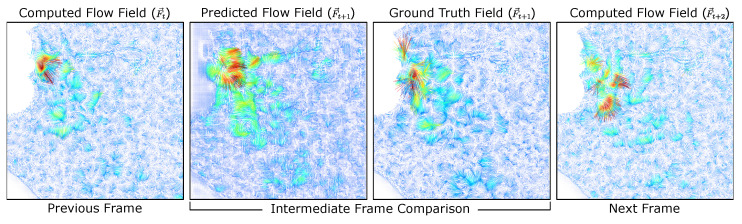
Flow field interpolation. Given the current and next frames at times (*t*, t+2), the predicted frame is compared to the ground truth, both of which represent the intermediate flow at time (t+1).

By selecting alternate frames, we can now compare the predicted flow field ${\vec{F}}_{t+1}$ at time t+1 with the ground truth (optical flow result) at time t+1. A visualization of the model predicted flow field and its corresponding ground-truth are shown in Figure 10.

### 3.12. Exhale Measures and Unique Signatures

A of the prominent aim of this work was to provide an accurate method for isolating and measuring exhale behaviors to identify behavioral differences in natural breathing between subjects. To validate the results generated from the model predicted flow fields, we generate exhale waveforms that represent the magnitude of observable flow for the recorded sequence. First, we illustrate the result of using the model to isolate and extract exhale behaviors compared to the result provided by using optical flow. From this, we obtain exhale signals with less noise. For all signals, the results are processed using three primary filters: (1) outlier removal outside of 1.5 standard deviation, (2) Savitzky–Golay with a window size of 9 samples, and (3) min-max normalization to the interval [0,1].

**Figure 11:**
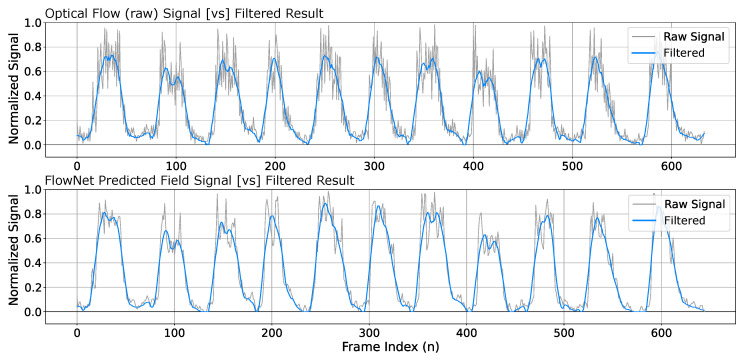
Optical flow (raw) versus model predicted flow field signal. FieldNet predicted flow fields contain a high signal-to-noise ratio that provides an implicit filtering of flow fields. The direct optical flow result (**top**) is compared with the model generated flow result (**bottom**).

Due to exhale behaviors rapidly dissipating within the thermal image sequences, the optical flow must be capable of identifying these flow characteristics compared to the noise floor. This results in the optical flow fields containing a significant amount of noise. We address this by utilizing FieldNet to predict flow images based on the original signal generated from the flow fields computed using optical flow. When the newly generated flow fields are predicted from the model, the resulting signal is significantly provides a better representation of the exhale behaviors with a better noise tolerance. Figure 11 illustrates the difference between the raw optical flow signal versus the signal generated from the FieldNet field prediction and demonstrates the resulting higher signal-to-noise ratio produced by the predictions of the model.

### 3.13. Exhale Episode Anomaly Detection

Capturing open-air behaviors as the foundation of a pulmonary diagnostic requires pre-processing and dataset normalization that can standardize observed respiratory behaviors. This is challenging due to the natural variance observed from turbulent exhale behaviors. However, while open-air turbulent flows contain per-exhale variance, they also exhibit clear patterns unique to each subject. These unique patterns demonstrate how natural breathing behaviors can be captured and exhibit the basis for furthering our understanding of pulmonary behavior. Therefore, we present a method for processing observed exhale waveforms to identify the unique characteristics of individualized behaviors and generate an anomaly score model to measure anomalous respiratory behaviors. We implement this by introducing two new models (1) an autoencoder model to reduce turbulent flow noise and (2) an anomaly prediction model. By creating a standardized set of normal or healthy classified behaviors used to train an anomaly score model, subsequent subject respiratory patterns can be evaluated through a trained convolutional model.

### 3.14. Exhale Segmentation

**Figure 12:**
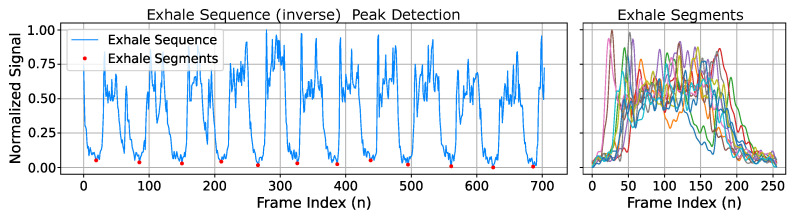
Exhale sequence peak detection and exhale segment extraction. Each recorded sequence is subdivided into individual exhales based on the minimal of each exhale. These are then consolidated into exhale segments (uniquely colored) used to train the filtering and anomaly detection models.

To evaluate anomalies within recorded exhale flow sequences predicted by FieldNet, we present an automated analysis for segmenting exhales and scoring each based on a trained anomaly model. Flow sequences are translated into continuous time-series sequences to isolate individual exhale episodes through peak detection. This converts each sequence from a continuous waveform into a collection of individual exhales as shown in Figure 12. Each exhale segment is then resampled to match the input of the pre-processing autoencoder and anomaly networks with input size of n=256.

### 3.15. Filtering Model

**Figure 13:**
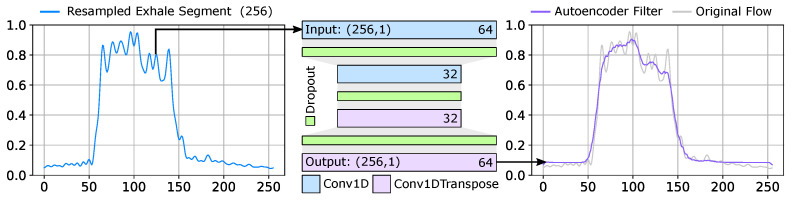
Segment filtering model. An autoencoder is employed as a noise reduction method to eliminate the high variance within each exhale segment caused by turbulent flow field magnitudes.

Turbulent open-air flow captured within each exhale exhibits a significant variance. While analytical noise removal can be applied to reduce these behaviors, they typically maintain anomalous spikes due to flow field variance. To address this, we use a one-dimensional filtering autoencoder to capture the key behavioral characteristics of the recorded exhale datasets. This requires a training dataset composed of individual exhale segments of length (256,1), where the expected value output is 1-to-1 with the provided input. The architecture of the autoencoder is presented in Figure 13. The model is composed of four layers (Conv1D, Conv1DTranspose) with rectified linear (relu) activation, kernel size k=7, trained using the Adam [69] optimizer with learning rate of $1.0\times 10^{-3}$. This model generates a filtered segment used for the anomaly detection model.

### 3.16. Anomaly Model

**Figure 14:**
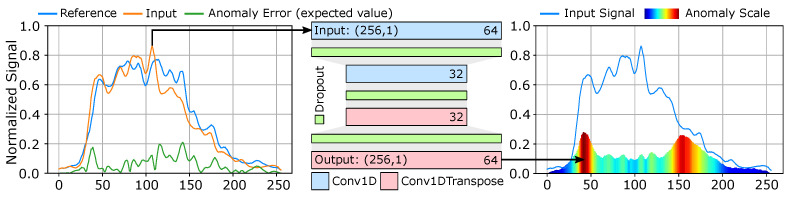
Anomaly model. Exhale instances are used to generate anomaly error waveforms by computing the absolute difference between the instance and the provided reference. This anomaly waveform is then used as the expected training value for the output of the network.

The aim of the proposed diagnostic is to contribute to the existing set of tests that can be performed to identify unique traits and possible anomalous behaviors within recorded breathing patterns. To provide the basis for model that can estimate anomalous behavior [70] within each exhale segment, we employ an *idealized* reference training set composed of instances from standardized exhale behaviors. These are exhales that exhibit the most regular behavior captured from subjects. This distinction is made on a simple health versus subject with pulmonary condition basis. To construct this the training set, we select (n=61) individual exhale segments $f{\left(t\right)}_{Ref}$ and a cross-mixed set of (m=103) exhale segments $f{\left(t\right)}_{Input}$ that contain both regularized and anomalous signatures for a total training set of n·m=6283. From these two sets, we form an error set by computing the absolute difference from each reference and input test sequence: ${e\left(t\right)=|f\left(t\right)}_{Ref}-f{\left(t\right)}_{Input}|$. This results in an (n·m) training dataset size, with each instance is defined as ($x_{train}=f{\left(t\right)}_{Input}$, $y_{train}=e\left(t\right)$) and the predicted value e(t) represents the anomaly error of the sequence. This provides a model that can predict the relative error between new exhale sequences and the reference set used to train the model. This model operates on the fixed length sequences of shape (256,1) and is shown in Figure 14. The model uses rectified linear (relu) activation, kernel size k=7, trained using the Adam [69] optimizer with learning rate of $1.0\times 10^{-3}$. Predicted error metrics are then presented as an anomaly scale that identifies unique traits of input exhale segments.

## 4. Results

Validation of the proposed method was performed using the data collected from 20 patients which provided twenty recordings of observable exhale flow. Each sequence contains 6–18 individual exhale episodes. These image sequences are provided to the processing pipeline that includes: (1) automated tracking, (2) localized flow field generation, (3) generation of the 1D exhale waveforms, and (4) anomaly detection. These results demonstrate the capability of the proposed method for capturing detailed natural exhale behaviors. This provides insight into how the natural breathing patterns of each subject result in different waveform characteristics that can be used to assist in the diagnostic process. By further evaluating the differences in these generated waveforms, anomalous behavioral analysis can be applied to identify potential variance in pulmonary function.

### 4.1. Localized Exhale Flow Prediction

**Figure 15:**
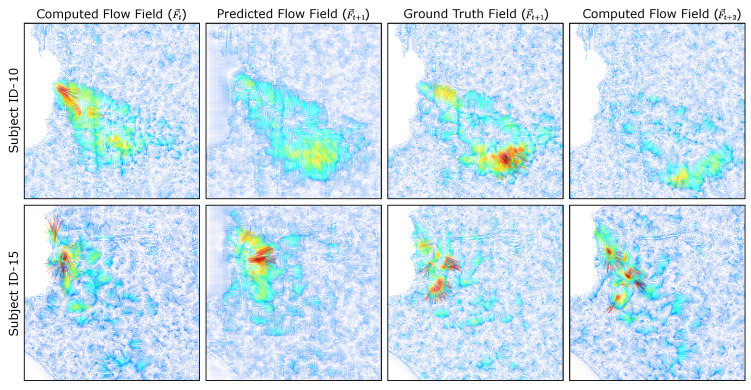
Multi-subject flow field interpolation. The interpolated result for arbitrary selected frames containing exhale flows are illustrated for three subjects. The ground truth field ${\vec{F}}_{t+1}$ is provided by the computed optical flow and compared to the predicted field ${\vec{F}}_{t+1}$. Color represents flow magnitude.

The prediction of the trained model obtains flow characteristics within the spatial distribution, magnitude, and localized behaviors. In various instances, including those visualized in Figure 15, the spatial distribution of the flow vectors represents an estimated approximation of the ground truth; however, the distribution of the vectors with the largest magnitudes may not directly correlate. Additionally, due to the zero regions introduced by the facial masking, the model has residual non-zero vector values that are estimated. While these vectors have a relatively small magnitude, they still contribute to error within the measurement of the exhale flow behavior.

### 4.2. Individualized Exhale Behaviors

**Figure 16:**
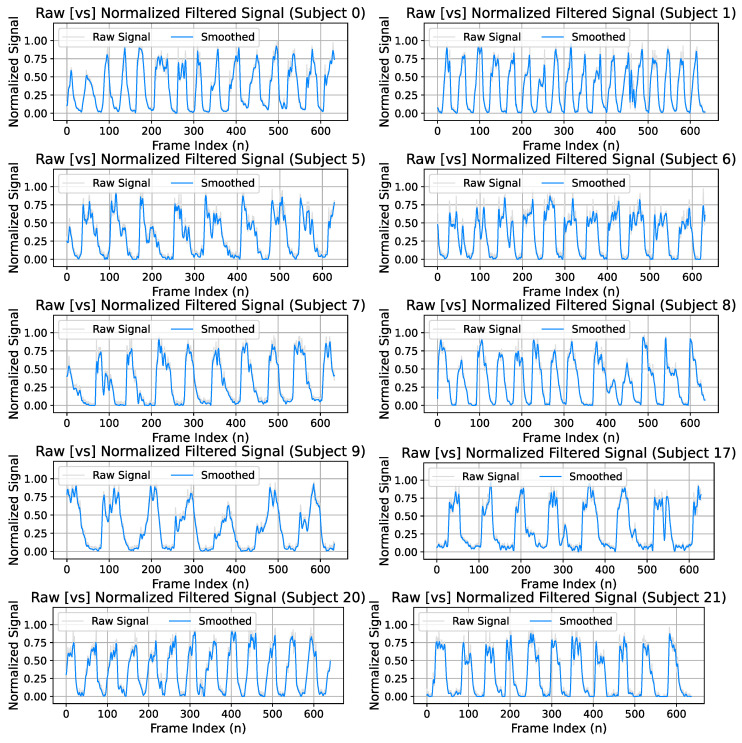
Select subject sequences (1–10). Each plot represents a collection of 500 flow frames that are used to generate expiatory waveforms. Each provides a unique subject exhale behaviors.

One of the primary objectives of this diagnostic is to provide a method for directly capturing exhale airflow behaviors without imposing any constraints on the subject. By eliminating the need for tubes and wearable solutions, the patient can resume subconscious breathing behaviors that are typically modified by exerting effort trying to maintain the constraints of the diagnostic system. This allows for the natural behaviors of each subject to be captured within the generated exhale waveform. This results in unique waveform characteristics for each subject including breathing rate, breath duration, and secondary metrics that can be obtained such as flow rate and volume. The generated exhale waveforms for select subjects have been presented within Figure 16. Each plot represents the recorded sequence waveform after processing. Each recording was performed for approximately 25 [s] for a total of 650 frames (samples) per waveform.

### 4.3. Anomaly Model Predictions

**Figure 17:**
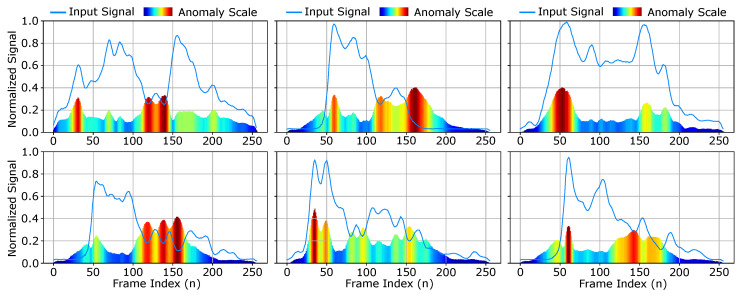
Anomaly results for select exhale segments. The input waveform is illustrated with the anomaly scale color mapped by magnitude. The regions of the waveform that illustrate anomalous behaviors result in higher predicted variance from the reference model.

The anomaly model predicts an objective estimation of how much the provided exhale segment diverges from the training set reference. This provides two useful purposes: (1) we can automate the process of scoring and isolating exhale segments that reach a given anomaly threshold and (2) we establish the basis for performing secondary diagnostics related to condition specific exhale behavioral traits. The anomaly measurements predicted for six randomly selected exhale segments are shown in Figure 17. This creates a unique form of behavioral analysis that provides an door opening opportunity to explore how the direct visualisation of exhale behaviors can contribute to the unique signatures of specific pulmonary conditions. While this approach provides this unique opportunity, there are still several challenges associated with visualized exhale flow imaging for pulmonary function analysis. These are covered in our discussion.

## 5. Discussion

The primary goal of the presented non-contact method is to capture, isolate, and measure the unique exhale traits that can be used to aid respiratory diagnostics. Similar to many of the different pulmonary diagnostic technologies, this approach has a mixture of advantages and disadvantages. As with all vision-based techniques for evaluating pulmonary behavior, there are a set of immediate challenges including, resolution, frame rate, occlusion, and overall accuracy. While these aspects of vision-based approaches present challenges in capturing respiratory behavior, it is one of the only methods for directly capturing exhaled CO_2_ flow at a distance. However, there are still factors that contribute to continued challenges with this approach. The first challenge is that there are physiological factors that may influence how recorded data reflects observable breathing patterns. While non-contact breathing can provide a unique look at pulmonary behavior, additional factors such as posture, mouth shape, and nasal blockages can contribute to variance within recorded observations. These factors partially contribute to the visualization obtained from the thermal camera, resulting in airflow differences. Experimental constraints can be used to minimize these factors, but they still may contribute to signal variance. The second is flow field estimation errors. There are instances where the face profile contributes to error within the generated flow sequences due to how the model will estimate flow contributions for null regions within the input flow. Other challenges are related to the formulation of the flow field model and signal generation. The FieldNet model can be used for two purposes: (1) interpolating between frames to increase the amount of 2D information translated into generated exhale waveforms and (2) to filter and isolate the exhale behavior to improve signal quality and reduce noise. The input of the model dictates that there must exist two flow states to predict an intermediary form. While this limits projective forecasting (or future state estimation) estimates, recordings of exhale behaviors are continuous within the collected data.

While this approach presents challenges associated with vision-based respiratory analysis, it also provides unique contributions towards the objective of obtaining natural, unrestricted breathing signals for diagnostic evaluation. This introduces the capability of identifying personalized characteristics of exhale behaviors by removing the conscious effort needed to maintain tube-based devices but also provides direct exhale flow observations that can be used to identify anomalies in regular breathing. By directly measuring the open airflow without impeding the subject’s breathing, we can obtain distinct patterns that can be linked to physiological effort, types of breathing, and traits belonging to specific pulmonary conditions. As part of our result evaluation, we identified unique waveform signatures from each subject, indicating that this could potentially be the case.

As a secondary extension of this approach, the proposed metho d can combined with existing respiratory monitoring devices that monitor deformations of the chest or diaphragm to capture inhale/exhale behaviors. This combination could be used to infer pulmonological traits from chest movements by correlating movement signals with airflow obtained from the direct CO_2_ flow visualization. By correlating visual flow data with existing methods for evaluating chest movement (ex. depth imaging, transducer belts), we can evaluate the relationship between chest/abdomen movement and exhaled CO_2_ airflow. This could enable the ability to aid in identifying the level of muscular activation indirectly from observable flow patterns, providing diagnostic synergy with existing solutions.

## 6. Future Developments

The presented work provides a basis for performing behavioral analysis of expiratory airflow to identify key traits that contribute to the unique pulmonary signatures. The objective of this approach was to illustrate how this novel method for capturing airflow through a non-contact method to provide effort-independent behavioral patterns. To further the utility of this approach, additional studies conducted with healthy subject versus subjects with known conditions must be performed to isolate how these behaviors contribute to unique identifiers that could be used to classify specific conditions. This requires the extension of the study to a larger subject sample size to identify the key trends that vary between breathing behaviors of one subject versus cross-subject patterns. Additionally, the models presented within this work provide a functional proof-of-concept but would benefit from larger training datasets to identify if condition-specific behavioral traits can be isolated. These additional factors would then provide the basis for comparing healthy subjects to subjects with specific pulmonary conditions.

## 7. Conclusions

In this work, we presented a method for pulmonary evaluation aimed at extracting the unique features of exhale behaviors from thermal image CO_2_ flow sequences. This provides a non-contact diagnostic for extracting exhale behaviors to obtain a natural and unrestricted measurement of expiratory airflow. Through the implementation of a convolutional neural network *FieldNet*, we evaluated how flow fields can be used to capture and isolate exhale behaviors from open-air recordings. Secondary convolutional networks were introduced and trained to reduce signal noise reduction and enable basic anomaly detection. This work provides the foundation for the classification of different pulmonary behaviors that can be identified through vision-based flow analysis. As part of ongoing research in direct exhale visualization and measurement, this approach provides an initial attempt at capturing individualized expiratory traits of natural breathing through CO_2_ flow analysis. While this approach does not directly distinguish between biological traces or provide direct validation of an exact pulmonary condition, it can be utilized to identify key individualistic behaviors that can assist in the evaluation of long-term trends of natural breathing and suggest additional screening based on detected anomalous behaviors. This provides alternative screening diagnostic that can be performed in between the larger context of pulmonary monitoring devices including wearables, existing PFT devices, and advanced scanning technologies to model natural breathing behaviors.

## Data Availability

The data presented in this study are available on request from the corresponding author.
